# Supplementary material for: Retrospective study of toxoplasmosis prevalence in pregnant women in Benin and its relation with malaria
Source: PLoS One. 2022 Jan 7;17(1):e0262018. doi: 10.1371/journal.pone.0262018 (PMC8741053; doi:10.1371/journal.pone.0262018)
Supplement: S1 Table — (DOCX) [file pone.0262018.s001.docx]

**Table S1: Effect of toxoplasmosis serological status and IPTp on malaria infection at delivery**

|  |  | **Pregnant women (n=948)** | | |
| --- | --- | --- | --- | --- |
| **Independent variables** | **Categories** | **Odds Ratio**^a^ | **[95% CI]** | ***P* value**^b^ |
| *T. gondii* positive serological status^c^ |  | 3.798 | [0.399; 36.189] | 0.246 |
| Number of IPTp-SP doses^d^ | [0,1] |  |  |  |
|  | [2,3] | 3.547 | [0.426; 29.542] | 0.242 |
| Toxoplasma serological status x IPTp-SP | Negative x 0-1 dose |  |  |  |
|  | Positive x 2-3 doses | 0.122 | [0.012; 1.229] | 0.074 |
| Adjustment variables: |  |  |  |  |
| Maternal age^e^ | [15–22]  [23–29]  [30–35] | 0.776  1.207 | [0.389; 1.550]  [0.594; 2.451] | 0.473  0.603 |
| Primigest *vs*. multigest women |  | 1.840 | [0.930; 3.643] | 0.080 |
| Living site | Akodeha  Ouedeme Pedah  Comé | 0.775  0.395 | [0.421; 1.425]  [0.218; 0.718] | 0.412  **0.002** |
| Maternal education^f^ | None  Partial primary  Complete primary  Beyond primary | 1.401  1.768  0.851 | [0.766; 2.561]  [0.742; 4.212]  [0.368; 1.968] | 0.274  0.198  0.707 |
| Number of visits (ANC + emergency)^g^ | [0–4]  [5,6]  [7–12] | 4.281  5.741 | [1.410; 13.001]  [1.880; 17.533] | **0.010**  **0.002** |
| Dry season |  | 1.109 | [0.683; 1.801] | 0.675 |
| Bednet possession |  | 0.953 | [0.552; 1.646] | 0.863 |

^a^: an Odds Ratio <1 shows a negative association between the variable and malaria infection whereas an Odds Ratio >1 shows a positive association.

^b^: significant P value <0.05 is in bold.

^c^: toxoplasmosis serological status defined at inclusion

^d^: number of IPTp-SP doses has been classified into 2 categories.

^e^: age has been divided into 3 periods.

^f^: maternal education was sequenced into 4 categories.

^g^: number of visits has been divided into 3 categories.

The logistic analysis was adjusted for maternal age, gravidity, living site, maternal education, number of visits, dry season and bednet possession.
