# Supplementary material for: Retrospective study of toxoplasmosis prevalence in pregnant women in Benin and its relation with malaria
Source: PLoS One. 2022 Jan 7;17(1):e0262018. doi: 10.1371/journal.pone.0262018 (PMC8741053; doi:10.1371/journal.pone.0262018)
Supplement: S2 Table — (DOCX) [file pone.0262018.s002.docx]

**Table S2: Differences in anti-*Pf*AMA1 IgG levels between inclusion and delivery**

|  |  | **Pregnant women (n=667)** | | |
| --- | --- | --- | --- | --- |
| **Independent variables** | **Categories** | **Coef**^a^ | **[95% CI]** | ***P* value**^b^ |
| *T. gondii* positive serological status^c^ |  | -0.198 | [-0.381; -0.014] | **0.035** |
| Malaria infection during follow-up^d^ |  | 0.098 | [0.436; 0.429] | **0.016** |
| Adjustment variables: |  |  |  |  |
| Maternal age^e^ | [15–22]  [23–29]  [30–35] | -0.061  -0.377 | [-0.311; 0.189]  [-0.641; -0.113] | 0.633  **0.005** |
| Primigest *vs*. multigest women |  | -0.045 | [-0.322; 0.232] | 0.750 |
| Living site | Akodeha  Ouedeme Pedah  Comé | 0.190  -0.861 | [-0.051; 0.431]  [-1.073; -0.646] | 0.123  **<0.001** |
| Maternal education^f^ | None  Partial primary  Complete primary  Beyond primary | -0.233  0.129  -0.242 | [-0.474; 0.007]  [-0.232; 0.491]  [-0.532; 0.049] | 0.058  0.483  0.103 |
| Number of visits (ANC + emergency)^g^ | [0–4]  [5,6]  [7–12] | -0.120  -0.173 | [-0.485; 0.245]  [-0.542; 0.197] | 0.520  0.359 |
| Dry season |  | 0.062 | [-0.121; 0.246] | 0.504 |
| Bednet possession |  | 0.008 | [-0.194; 0.210] | 0.941 |
| Number of IPTp-SP doses^h^ | [0,1]  [2,3] | -0.007 | [-0.458; 0.444] | 0.976 |

^a^: a coefficient <0 shows a negative association between the variable and maternal anti-*Pf*AMA1 IgG levels whereas a coefficient >0 shows a positive association.

^b^: significant P value <0.05 is in bold.

^c^: toxoplasmosis serological status defined at inclusion, 354 women were positive.

^d^: malaria infection was defined by at least one positive TBS during pregnancy (follow-up or delivery); 283 women had at least one malaria infection during the follow-up.

^e^: age has been divided into 3 periods.

^f^: maternal education was sequenced into 4 categories.

^g^: number of visits has been divided into 3 categories.

^h^: number of IPTp-SP doses has been classified into 2 categories.

The logistic analysis was adjusted for maternal age, gravidity, living site, maternal education, number of visits, dry season, bednet possession and number of IPTp-SP doses.
