# Supplementary material for: Retrospective study of toxoplasmosis prevalence in pregnant women in Benin and its relation with malaria
Source: PLoS One. 2022 Jan 7;17(1):e0262018. doi: 10.1371/journal.pone.0262018 (PMC8741053; doi:10.1371/journal.pone.0262018)
Supplement: Attachment — Submitted filename: Response to Reviewers.pdf [file pone.0262018.s003.pdf]

UMR 261 – Mère et enfant en milieu tropical
Directeur : Dr André Garcia

Dear members of the PLOS ONE Editorial Board,

Please find enclosed the revised version of our manuscript PONE-D-21-15174 entitled "Retrospective sero-epidemiological study of toxoplasmosis during pregnancy in Benin and potential relationships with malaria" in its originally submitted version, by Magalie Dambrun, Célia Dechavanne, Nicolas Guigue, Valérie Briand, Tristan Candau, Nadine Fievet, Murielle Lohezic, Saraniya Manoharan, Nawal Sare, Firmine Viwami, François Simon, Sandrine Houzé, Florence Migot-Nabias, that we like to be reconsidered for publication.

Please, also find with this letter, the detailed list of our responses to the editors and reviewers.

Thank you for your consideration.

Yours sincerely,

Magalie Dambrun, for the authors

Corresponding author:

Magalie Dambrun
UMR 261 MERIT
Faculté de Pharmacie de Paris
4 avenue de l'Observatoire
75006 Paris
France

Tel: +33 1 70 64 94 34
E-mail: magalie.dambrun@u-paris.fr

Journal requirements

1. Please ensure that your manuscript meets PLOS ONE's style requirements, including those for file naming. The PLOS ONE style templates can be found at https://journals.plos.org/plosone/s/file?id=wjVg/PLOSOne_formatting_sample_main_body.pdf[3] and https://journals.plos.org/plosone/s/file?id=ba62/PLOSOne_formatting_sample_title_authors_affiliations.pdf[4]

2. Please provide additional details regarding participant consent. In the ethics statement in the Methods and online submission information, please ensure that you have specified what type you obtained (for instance, written or verbal, and if verbal, how it was documented and witnessed). If your study included minors, state whether you obtained consent from parents or guardians. If the need for consent was waived by the ethics committee, please include this information.
The informed consent was written; this has been added line 258[1].

3. We note that the grant information you provided in the Funding Information and Financial Disclosure sections do not match.
When you resubmit, please ensure that you provide the correct grant numbers for the awards you received for your study in the Funding Information section.
There was no "Funding information" section in the original version of the manuscript and we had incorrectly inserted the financial information in the Acknowledgments section. In the revised version, we have implemented this section and corrected some inaccurate mentions lines 497-500.

4. Thank you for stating the following in the Acknowledgments Section of your manuscript:
We thank the Institut de Médecine et d'Epidémiologie Appliquée (IMEA) for financial support (grant 0602DIRmba).
As specified in point 3, IMEA provided some financial support, and as such, it must be mentioned in the « Funding information » section rather than in the Acknowledgments section.

We note that you have provided funding information that is not currently declared in your Funding Statement. However, funding information should not appear in the Acknowledgments section or other areas of your manuscript. We will only publish funding information present in the Funding Statement section of the online submission form.
We apologize for the confusion made in the financial sections and hope that this is now clarified.

Please remove any funding-related text from the manuscript and let us know how you would like to update your Funding Statement.
Currently, your Funding Statement reads as follows:
FMN : grant 0602DIRmba; Institut de Médecine et d'Epidémiologie Appliquée (IMEA); [https://www.imea.fr[5]https://www.imea.fr[5]; the funder in this study
NF : contract number 200889; European Union Framework 7; https://cordis.europa.eu/project/id/200889/]; the funder had a role of data collection in this study

Please include your amended statements within your cover letter; we will change the online submission form on your behalf.
Please correct the funding statement as follows:
FMN : grant 0206dDIR01; Institut de Médecine et d'Epidémiologie Appliquée (IMEA); [https://www.imea.fr]; the funder had a role in the acquisition of toxoplasmosis assay reagents.
NF : contract number 200889; European Union 7th Framework ; [https://cordis.europa.eu/project/id/200889]; the funder had a role in obtaining data on malaria.

[1] Numbered lines refer to the revised version of the manuscript with the changes visible (with track changes).

Reviewer's Responses to Questions

1. Is the manuscript technically sound, and do the data support the conclusions?
The manuscript must describe a technically sound piece of scientific research with data that supports the conclusions. Experiments must have been conducted rigorously, with appropriate controls, replication, and sample sizes. The conclusions must be drawn appropriately based on the data presented.
Reviewer #1: Yes Reviewer #2: Partly

2. Has the statistical analysis been performed appropriately and rigorously?
Reviewer #1: Yes Reviewer #2: I Don't Know

3. Have the authors made all data underlying the findings in their manuscript fully available?
The PLOS Data policy [7] requires authors to make all data underlying the findings described in their manuscript fully available without restriction, with rare exception (please refer to the Data Availability Statement in the manuscript PDF file). The data should be provided as part of the manuscript or its supporting information, or deposited to a public repository. For example, in addition to summary statistics, the data points behind means, medians and variance measures should be available. If there are restrictions on publicly sharing data e.g. participant privacy or use of data from a third party those must be specified.
Reviewer #1: No Reviewer #2: Yes
Concerning the data on malaria obtained within the framework of the STOPPAM project, the agreed rule is to contact the program referents for any agreement on the use of the information. On the other hand, the data generated on toxoplasmosis are now available on the site https://osf.io/. Information is mentioned lines 503-509.

4. Is the manuscript presented in an intelligible fashion and written in standard English?
PLOS ONE does not copyedit accepted manuscripts, so the language in submitted articles must be clear, correct, and unambiguous. Any typographical or grammatical errors should be corrected at revision, so please note any specific errors here.
Reviewer #1: Yes Reviewer #2: Yes

5. Review Comments to the Author
Please use the space provided to explain your answers to the questions above. You may also include additional comments for the author, including concerns about dual publication, research ethics, or publication ethics. (Please upload your review as an attachment if it exceeds 20,000 characters)

Reviewer #1

This is an interesting paper providing insights into the prevalence of toxoplasmosis in pregnant women and the incidence of congenital toxoplasmosis in Benin. The study is well-conducted and the paper reads very well. However, I have some important comments and some questions remain to be addressed.

First, the title is not self-explanatory and does not fully reflect the content of the paper. Maybe try: Retrospective study of prevalence of toxoplasmosis in pregnant women in Benin and its relation with malaria diagnosis at delivery?
We partially agree with the modification proposed by the reviewer, as relations between toxoplasmosis and malaria have been investigated as well during pregnancy (with the number of malaria infections) as at delivery (with the presence of malaria parasites in the placenta). As a compromise, we moved towards “Retrospective study of toxoplasmosis prevalence in pregnant women in Benin and its relation with malaria”.

Results:

Line 161: the occurrence of a positive (result) for the first assay and negative for the second, is not straightforward, as it is mentioned (line 151) that only seronegative women were re-tested ate delivery. Please explain how this sequence of results could happen.
The methodological sequences have been clarified lines 152 & 160-167.

Line 206: what do you mean by depleted? Were these 49 samples negative when tested diluted to 1/3?
The dosing strategy has been formulated more explicitly lines 206 & 211-213.

Line 267: I dont share this viewpoint. The rate of seroconversion should be calculated on the seronegative only. Seropositive at first serology cannot seroconvert.
This has been corrected, by removing the sentence referring to seroconversion taking into account seropositive women lines 277-279.

Line 271: it would be interesting here to convert this ratio into x/1000 live births, which is a widely rate used to compare incidence of diseases.
The requested precision has been provided line 282.

Line 278: it is not adapted to cite the gestational age here, especially as seropositive women were enrolled at an earlier stage (and the means are very close, 16.1 and 16.8 !). If seropositive had been enrolled at a later stage, it could have been an issue, as they might have acquired infection in early pregnancy (which would have been unnoticed as IgM were not tested).
We understand the Reviewer's remark, and we do not have any explanation for the lower gestational age at inclusion of women who are seropositive for toxoplasmosis. As Table 1 describes the context in which the women were included, we agree with the Reviewer's remark to keep only relevant information for the purpose of our study, and we have therefore removed this information from the text lines 288-290.

Line 293: were the prevalence of prematurity (1/12) and low birth weight (2/12) similar to that observed in the whole group of women?
Regarding the low number of women in the seroconversion group, it was decided not to present a statistical analysis in the manuscript. However, in response to the Reviewer, comparisons have been made below, and the following tables do not show any differences in prevalence of prematurity or low birth weight between seroconversion women and all women or negative women only or positive women only.

Prematurity

	term	premature	Total
all negative/positive	793	87	880
seroconversion	10	1	11
Total	803	88	891
		Fisher's exact	1

Low birth weight

	>2,5kg	<2,5kg (lbw)	Total
all negative/positive	745	100	845
seroconversion	9	2	11
Total	754	102	856
		Fisher's exact	0,63

Prematurity

	term	premature	Total

seronegative women	397	43	440
seroconversion	10	1	11
Total	407	44	451
		Fisher's exact	1

Low birth weight

	>2,5kg	<2,5kg (lbw)	Total
seronegative women	376	47	423
seroconversion	9	2	11
Total	385	49	434
		Fisher's exact	0.358

Prematurity

	term	premature	Total
seropositive women	439	46	485
seroconversion	10	1	11
Total	449	47	496
		Fisher's exact	1

Low birth weight

	>2,5kg	<2,5kg (lbw)	Total
seropositive women	410	56	466
seroconversion	9	2	11
Total	419	58	477
		Fisher's exact	0,631

Line 302: it is very surprising that IgM were detected in only 1/12 patients. As it is the hallmark of *Toxoplasma* acute infection, it casts doubt on the results of IgG. It cannot be excluded that the technique used for the first sample (Bioplex) lacks sensitivity. Were these samples re-analyzed using the WB Toxo IgGII to confirm that they were indeed negative at the beginning of pregnancy? This could also explain why ultrasound surveillance did not detect any abnormalities.
By contrast to the authors hypothesis (lines 367-70), it is unlikely that long-term storage of sera led to an alteration of antibody, as banks of sera are widely used to evaluate new serological assays with no such issue. Besides, infection with no IgM is a rare event (see Fricker-Hidalgo, JCM 2013). Or maybe the authors wanted to cite this paper but linked another one (ref #37)?
PW is probably not a seroconversion, as there is no move in antibody dosages, and should be probably excluded.
The authors report one infected neonate, following a maternal infection at around 4 months of pregnancy, it is all the more strange that no ultrasonographic signs were detected, as congenital infection at that stage is symptomatic. This should be discussed.
We thank the Reviewer for his/her insight and have provided clarifications that better validate these results. In particular, for the 12 women suspected of seroconversion, the baseline serology, determined for the first time with the Bioplex technique, was repeated (at the same time as the end of study sample) by the Platelia technique, to result in the same negative result. This is explained in the revised version (lines 152 & 160-167). Nevertheless, the WB Toxo II IgG was not used to confirm that these samples were negative at the beginning of pregnancy.
Otherwise, we confirm the correct use of references 36 (Fricker-Hidalgo H *et al*. 2013) and 37 (Arthur-Mensah Jnr R *et al*. 2016) with the following comments on lines 388-390: « The absence

(except in one case) of specific anti-*T. gondii* IgM may be explained by their lability, i.e., transient or short time presence, during the specific IgG positivation period [36]. However, an absence of specific IgM to toxoplasmosis is not uncommon, with no precise explanation provided [37]. ». On the other hand, we have removed the notion of instability of IgM linked to a long storage of plasma at -20°C, which does not seem plausible to the reviewer, whom we thank for sharing his/her experience with us.
We assume that the Reviewer refers to PW1 when he mentions that "PW is probably not a seroconversion ". In fact, there is no dosage with a strictly positive result during the follow-up of this patient. However, that were the results of the IgG avidity test on successive measurement points that led to estimate that there had been seroconversion.
Lastly, to strengthen our hypothesis on a role of SP in the prevention of serious sequelae linked to a maternal infection around the 4th month of pregnancy, we have reformulated differently the arguments presented in lines 422-427.

Table 4: were *Pf*AMA1 antibody dosages at inclusion and at delivery paired (same patient)? If not it seems difficult to interpret the decrease.
Thanks to the Reviewer for this relevant question. In the analysis presented in Table 4, the patients are not paired because we are not analyzing the decrease. We are just analyzing the levels at inclusion and at delivery. Because of this question, we have conducted an additional analysis on the decrease, presented in a new Supplementary Table (contributions in Material and methods lines 249-251 and in Results lines 346-351). The decrease in paired individuals is still significant meaning that women seropositive for *T. gondii* have lower levels of anti-*Pf*AMA1 IgG at delivery and they also produced less anti-*Pf*AMA1 IgG during pregnancy.

How was congenital infection diagnosed? Was IgM assessed in the cord blood? Only 2 infants had WB profiles different from their mother, so it is important to know how diagnosis was made or excluded.
Details on the diagnosis of congenital infection have been added lines 189-191 & 280-281.

Fig. 3&4: use preferably the same color legend. In Fig.4, bars should be grouped in each category.
The color legends have been harmonized for Figures 3 and 4.

Discussion:
Line 383: Investigating the impact of IPTp-SP on *T. gondii* infection: this sentence should appear in the objectives, at the beginning of the manuscript.
This notion has been added line 115.

Lines 397-401: should be deleted, as this trial cannot support, nor be compared to two intakes of sulfadoxine-pyrimethamine. Indeed, it was a clinical trial comparing sulfadiazine-pyrimethamine versus spiramycine, thus did not address the issue of treating or nor
treating. Furthermore, threatment was given daily.
Following the remark of the Reviewer, amendments were made to this part (lines 422-427), that we preferred to keep rather than delete.

Line 432: new antiparasitic compounds with anti-*T. gondii* and anti-*P. falciparum* cross-species efficacy could soon be defined: such what? Antiparasitic drugs already target the same pathways. Maybe this sentence could be rephrased, if the authors rather think of immune cross-reactivity (vaccine development or immunomodulation therapy?).
We agree with the Reviewer that this sentence has no place here and we have deleted it.

How do the authors explain that *Toxoplasma* seropositivity had a preventive effect on malaria infection at delivery, but no overall effect during follow-up (table 3)?
The association between *T. gondii* serological status and *P. falciparum* infection during pregnancy is not significant (OR=0.835 [0.626; 1.116], p value=0.223) but the negative value of the OR (<1) goes in the same direction as what is observed at delivery. We can assume that the malaria infection measured at delivery (on maternal peripheral blood and/or placental blood) may

correspond to a cumulative measurement of the events during pregnancy, in particular with the sequestration of *P. falciparum* parasitic forms in the placenta.

Minor points:
Line 62: prefer inversely correlated to the age of pregnancy at maternal infection
Line 72: sulfadoxine is no more available for the treatment of toxoplasmosis.
Changes have been made.

Reviewer #2

General comments
The manuscript presented *Toxoplasma* seroepidemiology and malaria infection in a large cohort of pregnant women in a region of Benin.
The study is interesting and well-written. It showed the epidemiology of congenital toxoplasmosis in a region where is applied an intermittent preventive treatment against malaria during pregnancy using a sulfadoxine-pyrimethamine (SP) combination (IPTp-SP).
However, based on the results of this observational study in a specific population, the authors tried to find a relation between toxoplasmosis and malaria.

Specific comments
Title
The data presented in the study did not show any relation between toxoplasmosis and malaria during pregnancy. In consequence, could the authors modify the title by suppressing potential relationship?
We agree that the initial wording of the title could imply the investigation of relations between the two infections that are toxoplasmosis and malaria, which is indeed not the case. We have reformulated the title, where it appears more explicitly that it is the seroprevalence of toxoplasmosis that is examined with regard to several malaria parameters. Thereby, the new title is: "Retrospective study of toxoplasmosis prevalence in pregnant women in Benin and its relation with malaria".

Abstract
Lines 37-39: Could the authors specify IgG are anti- *P. falciparum* Apical Membrane Antigen 1?
This has been specified.

Mat & Meth
Lines 163-164: Could the authors reconsider this sentence?
According to the reviewer, this assay has been developed and been used to detect low titers of specific anti-Toxoplasma IgG. It is not a screening test, as some assays using a limited number of parasite antigens.
The sentence has been rephrased.

Results
Toxoplasmosis seroprevalence, seroconversion and CT
Could the authors give here or in Seroconversion maternal group and infants the % of CT among infants whose mothers had an infection during pregnancy?
This information is given in the first paragraph of the Results section.

Association between *T. gondii* serological status and *P. falciparum* infection during pregnancy
Lines 320-323: The OR is low 0.531 (< 2 fold fewer risks for *Toxoplasma* seropositive pregnant women to have malaria at delivery). Could the authors specify this in the section Discussion?
The association between *T. gondii* serological status and *P. falciparum* infection during pregnancy is not significant (OR=0.835 [0.626; 1.116], p value=0.223) but the negative value of the OR (<1) goes in the same direction as what is observed at delivery. We can assume that the malaria infection measured at delivery (on maternal peripheral blood and/or placental blood) may

correspond to a cumulative measurement of the events during pregnancy, in particular with the sequestration of *P. falciparum* parasitic forms in the placenta.

Table 3: Could the authors shortly present in the text the results of malaria risks according to the number of visits?
The number of diagnosed malaria infections increased with the number of visits, and more precisely with the number of emergency visits (rather than with antenatal programmed visits), in link with symptoms motivating a medical visit. In fact, the items considered in Table 3 are adjustment variables, which contribute in the multivariate analysis, to reinforce a direct link between *T. gondii* serological status and malaria, independently of variables (such as the number of visits) interfering with malaria (but not with toxoplasmosis). It would be confusing to comment the results inherent to all adjustment variables, so we chose to limit comments to what is added in lines 334-338.

Discussion
Lines 350-351: The authors should add that the lack of opthalmological examination in infants in their study could lead to misdiagnosing CT cases.
This has been added lines 370-371.

Lines 410-412: Could the authors give the % of homology between the proteins of *T. gondii* and *P. falciparum*.
The precision has been made line 437.

The authors should consider that the tendency to observe a lower malaria infection at delivery occurred in a population of women treated with sulfadoxine-pyrimethamine (SP) combination. To support a relation between toxoplasmosis and malaria such a study should be done in non-treated pregnant women and, or the general population. Indeed, we could not exclude that such treatment affected the risk of malaria between *Toxoplasma* negative and seropositive pregnant women. To support this last hypothesis, the rate of malaria was not different between *Toxoplasma* negative and seropositive women at the beginning of pregnancy (Figure 3).
We totally agree with the Reviewer #2. We cannot exclude that SP treatment affect the risk of malaria between *Toxoplasma* negative and seropositive pregnant women. In order to ensure about the real effect of *Toxoplasma* serology on malaria infection, we included the number of IPTp doses in the multivariate analysis (Table 3 and Table 4). We found that a high number of IPTp doses (2 to 3 doses) has a tendency to decrease malaria infections during the follow-up (OR=0.642 [0.379; 1.088], p value=0.100). Because this variable is included in the multivariate model, its effect on malaria infection is taken into account and cannot explain the association between the *Toxoplasma* serology and malaria infection at delivery.

To ensure about our conclusion in the article, we added another analysis deciphering the effect into the four groups:

(i) Toxoplasma negative / no or one dose of IPTp,
(ii) Toxoplasma negative / two or three doses of IPTp,
(iii) Toxoplasma positive / no or one dose of IPTp and
(iv) Toxoplasma positive / two or three doses of IPTp

and malaria infection during the follow-up or at delivery. We performed a logistic model with the interaction term (*Toxoplasma* x IPTp). In the following table, the Reviewer can see that there is no synergic effect of *Toxoplasma* and IPTp on malaria at delivery. The results are stronger for malaria during the follow-up (OR=0.922 [0.408; 2.082], p value=0.846).

Independent variables	Categories	Odds Ratio	*P* value	[95% CI]
T. gondii positive serological status		3,798	0,246	[0,399 ; 36,189]
Number of IPTp doses	[0,1] (ref)	3,547	0,242	[0,426 ; 29,542]
	[2,3]			
Toxoplasma x IPTp	negative x 0-1 dose (ref)			
	positive x 2-3 doses	0,122	0,074	[0,012 ; 1,229]
Maternal education	none (ref)			
	partial primary	1,401	0,274	[0,766 ; 2,561]
	complete primary	1,768	0,198	[0,742 ; 4,212]
	beyond primary	0,851	0,707	[0,368 ; 1,968]
Bednet possession at inclusion		0,953	0,863	[0,552 ; 1,646]
Primigavidae		1,840	0,080	[0,930 ; 3,643]
Age	[15,22] (ref)			
	[23,29]	0,776	0,473	[0,389 ; 1,550]
	[30,45]	1,207	0,603	[0,594 ; 2,451]
Number of visits	[0-4] (ref)			
	[5,6]	4,281	0,010	[1,410 ; 13,001]
	[7-12]	5,741	0,002	[1,880 ; 17,533]
Dry season at inclusion		1,109	0,675	[0,683 ; 1,801]
Living site	AK (ref)			
	CM	0,395	0,002	[0,218 ; 0,718]
	WP	0,775	0,412	[0,421 ; 1,425]

Finally, the data are old (2008-2010, more than ten years).

We agree with the Reviewer that the data are more than 10 years old and that their analysis can hardly lead to the delivery of public health prevention messages. However, to our knowledge, there is no study on toxoplasmosis during pregnancy in Benin that has involved such a large number of individuals as the almost 1000 women considered here. We therefore considered it relevant to conduct this retrospective serological study. In addition, this was a unique opportunity to correlate this information on toxoplasmosis with clinical, parasitological and immunological data on malaria.
