# Supplementary material for: Retrospective study of toxoplasmosis prevalence in pregnant women in Benin and its relation with malaria
Source: PLoS One. 2022 Jan 7;17(1):e0262018. doi: 10.1371/journal.pone.0262018 (PMC8741053; doi:10.1371/journal.pone.0262018)
Supplement: Attachment — Submitted filename: Dambrun et al_Response to Reviewers.pdf [file pone.0262018.s004.pdf]

UMR 261 – Mère et enfant en milieu tropical
Directeur : Dr André Garcia

Dear members of the PLOS ONE Editorial Board,

Please find enclosed the revised version of our manuscript PONE-D-21-15174R1 entitled "Retrospective study of toxoplasmosis prevalence in pregnant women in Benin and its relation with malaria" in its revised submitted version, by Magalie Dambrun, Célia Dechavanne, Nicolas Guigue, Valérie Briand, Tristan Candau, Nadine Fievet, Murielle Lohezic, Saraniya Manoharan, Nawal Sare, Firmine Viwami, François Simon, Sandrine Houzé, Florence Migot-Nabias, that we like to be reconsidered for publication.

Please, also find with this letter, the detailed list of our responses to the editors and reviewers.

Thank you for your consideration.

Yours sincerely,

Magalie Dambrun, for the authors

**Corresponding author:**
Magalie Dambrun
UMR 261 MERIT
Faculté de Pharmacie de Paris
4 avenue de l'Observatoire
75006 Paris
France

Tel: +33 1 70 64 94 34
E-mail: magalie.dambrun@u-paris.fr

## Responses to Reviewer #1

The authors have addressed most comments, but some clarifications are still needed.

About M&M of serology:
-line 160: do you mean that when the Platelia IgG assay was positive at the end of pregnancy for a woman previously diagnosed as seronegative, both sera (inclusion and end) were retested with Platelia IgG? This is still not clear.
-line 163: for the rare samples with confirmed discrepant results, do you mean negative with Bioplex and positive with Platelia at inclusion ? or samples at end of study that tested positive, then negative, with Platelia during the first and second run, respectively ?
The sentences have been modified in order to better explicit the methodology applied, namely for the plasma samples of the end of the study (lines 160-166 of the revised version 2 of the manuscript).

-line 174: qualitative should be removed, as the Platelia assay is a quantitative assay.
The Bio-Rad supplier's notice specifies that qualitative Platelia™ TOXO IgM allows a "qualitative detection of IgM anti-*Toxoplasma gondii* antibodies in serum or human plasma by immunoenzymatic method" (cited in text). Results are therefore classified into 3 categories (negative, doubtful, positive) according to an absorbance ratio. For this reason, and despite the Reviewer's request, we have not changed the terms of the line 176 of the v2 revised version of the manuscript.

Results:
-line 212: were any of these 49 women with a negative result obtained on diluted samples at inclusion, diagnosed seropositive at delivery? It should be mentioned, so that there is doubt regarding the 12 announced cases of seroconversion.
We understand that this aspect needs to be clarified. As already mentioned, the seronegativity at study exit could be confirmed for 105 out of 154 plasma samples whose equivalent plasma samples at inclusion were diluted 1/3. It was not possible to realize the assay on the remaining 49 plasmas at study exit because they were depleted (lines 212-213 of the v2 revised version). The arbitrary decision was taken to consider them negative, this decision having little impact on the seroprevalence rate, as specified in the lines 214-216 of the v2 version.
Nevertheless, we understand the concern of the Reviewer regarding the doubt that this may raise on potential non-diagnosed cases of seroconversion among the 49 women represented by these plasma samples. In order to answer it, details have been provided in two places of the manuscript:

- Results, part "Seroconversion maternal group and infants": for 7 out of these 12 women, plasma samples at inclusion were diluted 1/3 by the ToRC assay, and their negativity confirmed later using the Platelia Toxo IgG assay on non-diluted plasma samples (lines 304-306).
- Discussion: it was already mentioned that "the depletion in some samples at delivery contributed surely to an underestimation of the number of *T. gondii* seronegative women having seroconverted" (lines 373-375).

-line 269: precise n/N seronegative samples available at delivery
The precision has been made (lines 270-272).

-line 274: precise available plasma samples at delivery.

-line 275: if there were 12 mother-cord plasma samples, why were only 9 available for highlighting potential cases of CT ? It could be rephrased as from the 12 cases identified with seroconversion during pregnancy, mother and cord blood paired samples were available in only 9. For better understanding, please mention the results of Platelia Toxo IgM (0/9 positive samples ?) and of WB IgG/IgM, as described in M&M.
This part has been rephrased as recommended by the Reviewer (lines 277-280).

-line 277: regarding the rate of CT: 2/820 live births: does it mean that there has been 159 miscarriages? This should be discussed, as some fetal losses might have been due to unrecognized toxoplasmosis.
The birth count has been taken again, corrected and detailed in the results (lines 281-286). We namely added the following sentence: "Knowing that 19 births were twins, 163 deliveries remained unrecorded, for 816 women (i.e., 835-19) among the whole study group of 979 women. These 163 situations were distributed into 80 women lost to follow-up or having stopped the follow-up (49.1%), 35 stillbirths (21.5%), 32 miscarriages (19.6%) and 16 women whose pregnancy has been invalidated (9.8%)". For the attention of the Reviewer, the distribution of all different types of pregnancy outcomes according to the toxoplasmosis serological status at inclusion is detailed in the table attached here. It would be interesting to seek to clarify a possible imbalance in the cases of stillbirths and miscarriages, which seem to be more numerous in women seropositive for toxoplasmosis. Nevertheless, regarding stillbirths, placental biopsies were not suitable for a search of *T. gondii* by molecular methods, because formalin was used for histological examinations. We agree with the Reviewer that some of the stillbirths and/or miscarriages registered among women seronegative at inclusion may be attributable to toxoplasmosis contracted during pregnancy. We chose to not overload the manuscript and added only in the discussion (line 374) that both miscarriages and stillbirths may contribute to an underestimation of the number of seroconversion cases.

| Different types of pregnancy outcomes | **Toxo NEG** | **Toxo POS** | **Tech failure** | **TOTAL** |
|---|---|---|---|---|
| Normal childbirths | 392 | 419 | 5 | 816 |
| Stillbirths | 14 | 21 | | 35 |
| Invalidated pregnancies | 4 | 12 | | 16 |
| Women lost to follow-up | 33 | 33 | | 66 |
| Women having stopped the follow-up | 6 | 8 | | 14 |
| Miscarriages | 13 | 19 | | 32 |
| TOTAL | 462 | 512 | 5 | 979 |

-the table presented in the response to reviewer 2 should be shown, at least as supplemental material.
A supplemental Table has been made, which is cited in the manuscript on line 342. This Table is named S1 and the previous supplemental Table is renamed S2.

Discussion:
-line 367: the rate of seroconversion is 3.4%, not 1.4%, as calculated in the Results section.
In the first paragraph of the discussion (lines 362-363), we added under brackets the reason explaining why we employed the rate of seroconversion as expressed among the whole group of women (1.4%) rather than among the seronegative women (3.4%).

-line 383: I would like to insist that seroconversion with no IgM is a rare event, thus it not not uncommon, as written, and the sentence must be amended. The study by Fricker-Hidalgo reported 15 cases from 12 centers over 10 years, i.e. 15/4500 (0.3%) cases of seroconversion, which is far different from your results.
The sentence has been amended as recommended by the Reviewer (lines 394-395).

-lines 416-19: need to be rephrased. Maybe try Thus, although the sulfamide-pyrimethamine regimens were very different between this latter study and ours, it led to a reduced severity of the fetal sequelae, which is in agreement with the absence of ultrasonographic signs in our PW2 case despite a maternal infection having occurred at around 4 months of gestation.
We thank the reviewer for the sentence proposed, which has been integrated in the text (lines 429-432).

**Response to Reviewer #2**

The authors answered all the comments of the reviewer.

Minor comment
Lines 364-365: Ophthalmological follow-up (as serological follow-up) during the first year of life is sufficient to diagnose CT. Long-term ophthalmological follow-up is useful to diagnose ocular toxoplasmosis in newborns previously diagnosed with CT.
We modified the sentence based on the Reviewer's advice (lines 376-377).
